# Supplementary material for: Comparative multiomics analysis of cell physiological state after culture in a basket bioreactor
Source: Sci Rep. 2022 Nov 23;12:20161. doi: 10.1038/s41598-022-24687-4 (PMC9686226; doi:10.1038/s41598-022-24687-4)
Supplement: Supplementary file 1 — Supplementary Information 1. [file 41598_2022_24687_MOESM1_ESM.zip › raw data/Metabolomics raw data/5.MetKeggEnrichment/EG.vs.CG/EG.vs.CG_pos_KEGG_map/KEGG.map.html]

Pathway Enrichment


  

# The most enriched pathway terms of EG.vs.CG\_pos

Statistic method: hypergeometric test

FDR correction method: Benjamini and Hochberg

| MapID | MapTitle | Pvalue | AdjustedPv | x | y | n | N | EnrichDirect | GeneIDs |
| --- | --- | --- | --- | --- | --- | --- | --- | --- | --- |
| map00260 | Glycine, serine and threonine metabolism | 0.0513410639992919 | 0.642648490749756 | 3 | 3 | 30 | 79 | Over | Com\_389\_pos Com\_45\_pos Com\_629\_pos |
| map00220 | Arginine biosynthesis | 0.141187925998053 | 0.642648490749756 | 2 | 2 | 30 | 79 | Over | Com\_1117\_pos Com\_342\_pos |
| map00270 | Cysteine and methionine metabolism | 0.141187925998053 | 0.642648490749756 | 2 | 2 | 30 | 79 | Over | Com\_1683\_pos Com\_629\_pos |
| map04022 | cGMP-PKG signaling pathway | 0.141187925998053 | 0.642648490749756 | 2 | 2 | 30 | 79 | Over | Com\_13\_pos Com\_218\_pos |
| map04152 | AMPK signaling pathway | 0.141187925998053 | 0.642648490749756 | 2 | 2 | 30 | 79 | Over | Com\_13\_pos Com\_3667\_pos |
| map04211 | Longevity regulating pathway | 0.141187925998053 | 0.642648490749756 | 2 | 2 | 30 | 79 | Over | Com\_13\_pos Com\_3667\_pos |
| map04212 | Longevity regulating pathway - worm | 0.141187925998053 | 0.642648490749756 | 2 | 2 | 30 | 79 | Over | Com\_343\_pos Com\_3667\_pos |
| map04740 | Olfactory transduction | 0.141187925998053 | 0.642648490749756 | 2 | 2 | 30 | 79 | Over | Com\_13\_pos Com\_218\_pos |
| map00330 | Arginine and proline metabolism | 0.193510380718908 | 0.642648490749756 | 4 | 6 | 30 | 79 | Over | Com\_1683\_pos Com\_2706\_pos Com\_342\_pos Com\_45\_pos |
| map01100 | Metabolic pathways | 0.264039744015913 | 0.642648490749756 | 25 | 59 | 30 | 79 | Over | Com\_1117\_pos Com\_13\_pos Com\_1480\_pos Com\_15057\_pos Com\_16813\_pos Com\_1683\_pos Com\_1804\_pos Com\_2109\_pos Com\_218\_pos Com\_2321\_pos Com\_252\_pos Com\_2602\_pos Com\_2706\_pos Com\_3077\_pos Com\_342\_pos Com\_343\_pos Com\_3667\_pos Com\_389\_pos Com\_45\_pos Com\_4716\_pos Com\_486\_pos Com\_572\_pos Com\_6063\_pos Com\_629\_pos Com\_8189\_pos |
| map00030 | Pentose phosphate pathway | 0.379746835443038 | 0.642648490749756 | 1 | 1 | 30 | 79 | Over | Com\_16813\_pos |
| map00051 | Fructose and mannose metabolism | 0.379746835443038 | 0.642648490749756 | 1 | 1 | 30 | 79 | Over | Com\_3077\_pos |
| map00190 | Oxidative phosphorylation | 0.379746835443038 | 0.642648490749756 | 1 | 1 | 30 | 79 | Over | Com\_3667\_pos |
| map00250 | Alanine, aspartate and glutamate metabolism | 0.379746835443038 | 0.642648490749756 | 1 | 1 | 30 | 79 | Over | Com\_342\_pos |
| map00261 | Monobactam biosynthesis | 0.379746835443038 | 0.642648490749756 | 1 | 1 | 30 | 79 | Over | Com\_1683\_pos |
| map00430 | Taurine and hypotaurine metabolism | 0.379746835443038 | 0.642648490749756 | 1 | 1 | 30 | 79 | Over | Com\_342\_pos |
| map00471 | D-Glutamine and D-glutamate metabolism | 0.379746835443038 | 0.642648490749756 | 1 | 1 | 30 | 79 | Over | Com\_342\_pos |
| map00520 | Amino sugar and nucleotide sugar metabolism | 0.379746835443038 | 0.642648490749756 | 1 | 1 | 30 | 79 | Over | Com\_3077\_pos |
| map00524 | Neomycin, kanamycin and gentamicin biosynthesis | 0.379746835443038 | 0.642648490749756 | 1 | 1 | 30 | 79 | Over | Com\_342\_pos |
| map00600 | Sphingolipid metabolism | 0.379746835443038 | 0.642648490749756 | 1 | 1 | 30 | 79 | Over | Com\_5126\_pos |
| map00630 | Glyoxylate and dicarboxylate metabolism | 0.379746835443038 | 0.642648490749756 | 1 | 1 | 30 | 79 | Over | Com\_342\_pos |
| map00650 | Butanoate metabolism | 0.379746835443038 | 0.642648490749756 | 1 | 1 | 30 | 79 | Over | Com\_342\_pos |
| map00660 | C5-Branched dibasic acid metabolism | 0.379746835443038 | 0.642648490749756 | 1 | 1 | 30 | 79 | Over | Com\_342\_pos |
| map00730 | Thiamine metabolism | 0.379746835443038 | 0.642648490749756 | 1 | 1 | 30 | 79 | Over | Com\_3667\_pos |
| map00910 | Nitrogen metabolism | 0.379746835443038 | 0.642648490749756 | 1 | 1 | 30 | 79 | Over | Com\_342\_pos |
| map01200 | Carbon metabolism | 0.379746835443038 | 0.642648490749756 | 1 | 1 | 30 | 79 | Over | Com\_16813\_pos |
| map04024 | cAMP signaling pathway | 0.379746835443038 | 0.642648490749756 | 1 | 1 | 30 | 79 | Over | Com\_13\_pos |
| map04068 | FoxO signaling pathway | 0.379746835443038 | 0.642648490749756 | 1 | 1 | 30 | 79 | Over | Com\_13\_pos |
| map04122 | Sulfur relay system | 0.379746835443038 | 0.642648490749756 | 1 | 1 | 30 | 79 | Over | Com\_1683\_pos |
| map04150 | mTOR signaling pathway | 0.379746835443038 | 0.642648490749756 | 1 | 1 | 30 | 79 | Over | Com\_13\_pos |
| map04151 | PI3K-Akt signaling pathway | 0.379746835443038 | 0.642648490749756 | 1 | 1 | 30 | 79 | Over | Com\_13\_pos |
| map04216 | Ferroptosis | 0.379746835443038 | 0.642648490749756 | 1 | 1 | 30 | 79 | Over | Com\_8189\_pos |
| map04725 | Cholinergic synapse | 0.379746835443038 | 0.642648490749756 | 1 | 1 | 30 | 79 | Over | Com\_389\_pos |
| map04744 | Phototransduction | 0.379746835443038 | 0.642648490749756 | 1 | 1 | 30 | 79 | Over | Com\_218\_pos |
| map04923 | Regulation of lipolysis in adipocytes | 0.379746835443038 | 0.642648490749756 | 1 | 1 | 30 | 79 | Over | Com\_13\_pos |
| map04924 | Renin secretion | 0.379746835443038 | 0.642648490749756 | 1 | 1 | 30 | 79 | Over | Com\_13\_pos |
| map04931 | Insulin resistance | 0.379746835443038 | 0.642648490749756 | 1 | 1 | 30 | 79 | Over | Com\_1198\_pos |
| map05323 | Rheumatoid arthritis | 0.379746835443038 | 0.642648490749756 | 1 | 1 | 30 | 79 | Over | Com\_6063\_pos |
| map00400 | Phenylalanine, tyrosine and tryptophan biosynthesis | 0.553863857661326 | 0.761562804284323 | 2 | 3 | 30 | 79 | Over | Com\_16813\_pos Com\_2109\_pos |
| map00564 | Glycerophospholipid metabolism | 0.553863857661326 | 0.761562804284323 | 2 | 3 | 30 | 79 | Over | Com\_389\_pos Com\_486\_pos |
| map00760 | Nicotinate and nicotinamide metabolism | 0.553863857661326 | 0.761562804284323 | 2 | 3 | 30 | 79 | Over | Com\_343\_pos Com\_3667\_pos |
| map01523 | Antifolate resistance | 0.553863857661326 | 0.761562804284323 | 2 | 3 | 30 | 79 | Over | Com\_13\_pos Com\_218\_pos |
| map04742 | Taste transduction | 0.553863857661326 | 0.761562804284323 | 2 | 3 | 30 | 79 | Over | Com\_13\_pos Com\_218\_pos |
| map05231 | Choline metabolism in cancer | 0.553863857661326 | 0.761562804284323 | 2 | 3 | 30 | 79 | Over | Com\_389\_pos Com\_486\_pos |
| map04977 | Vitamin digestion and absorption | 0.668422903599453 | 0.86501787524635 | 3 | 6 | 30 | 79 | Over | Com\_343\_pos Com\_3667\_pos Com\_6063\_pos |
| map01230 | Biosynthesis of amino acids | 0.724144233131553 | 0.919106142051586 | 4 | 9 | 30 | 79 | Over | Com\_1117\_pos Com\_16813\_pos Com\_1683\_pos Com\_629\_pos |
| map00052 | Galactose metabolism | 1 | 1 | 1 | 2 | 30 | 79 | Over | Com\_1804\_pos |
| map00061 | Fatty acid biosynthesis | 1 | 1 | 1 | 2 | 30 | 79 | Over | Com\_11397\_pos |
| map00100 | Steroid biosynthesis | 1 | 1 | 1 | 2 | 30 | 79 | Over | Com\_6063\_pos |
| map00240 | Pyrimidine metabolism | 1 | 1 | 2 | 5 | 30 | 79 | Over | Com\_1480\_pos Com\_572\_pos |
| map00340 | Histidine metabolism | 1 | 1 | 2 | 5 | 30 | 79 | Over | Com\_342\_pos Com\_4716\_pos |
| map00380 | Tryptophan metabolism | 1 | 1 | 1 | 2 | 30 | 79 | Over | Com\_2109\_pos |
| map00480 | Glutathione metabolism | 1 | 1 | 3 | 7 | 30 | 79 | Over | Com\_15057\_pos Com\_342\_pos Com\_8189\_pos |
| map00750 | Vitamin B6 metabolism | 1 | 1 | 1 | 2 | 30 | 79 | Over | Com\_16813\_pos |
| map00860 | Porphyrin and chlorophyll metabolism | 1 | 1 | 1 | 2 | 30 | 79 | Over | Com\_342\_pos |
| map00980 | Metabolism of xenobiotics by cytochrome P450 | 1 | 1 | 1 | 2 | 30 | 79 | Over | Com\_5288\_pos |
| map04925 | Aldosterone synthesis and secretion | 1 | 1 | 1 | 2 | 30 | 79 | Over | Com\_3667\_pos |
| map05204 | Chemical carcinogenesis | 1 | 1 | 1 | 2 | 30 | 79 | Over | Com\_5288\_pos |
